# Succinate Activates EMT in Intestinal Epithelial Cells through SUCNR1: A Novel Protagonist in Fistula Development

**DOI:** 10.3390/cells9051104

**Published:** 2020-04-29

**Authors:** Dolores Ortiz-Masiá, Laura Gisbert-Ferrándiz, Cristina Bauset, Sandra Coll, Céline Mamie, Michael Scharl, Juan V. Esplugues, Rafael Alós, Francisco Navarro, Jesús Cosín-Roger, María D. Barrachina, Sara Calatayud

**Affiliations:** 1 Departamento de Medicina, Facultad de Medicina, Universidad de Valencia, 46010 València, Spain; M.Dolores.Ortiz@uv.es; 2 Departamento de Farmacología and CIBER, Facultad de Medicina, Universidad de Valencia, 46010 València, Spain; laura.gisbert@uv.es (L.G.-F.); juan.v.esplugues@uv.es (J.V.E.); dolores.barrachina@uv.es (M.D.B.); sara.calatayud@uv.es (S.C.); 3 Departamento de Farmacología, Facultad de Medicina, Universidad de Valencia, 46010 València, Spain; baupas@alumni.uv.es (C.B.); Sandra.Coll@uv.es (S.C.); 4 Department of Gastroenterology and Hepatology, University Hospital of Zurich, University of Zurich, 8091 Zürich, Switzerland; Celine.Mamie@usz.ch (C.M.); Michael.Scharl@usz.ch (M.S.); 5 Hospital Dr Peset—FISABIO, 46017 València, Spain; 6 Hospital Universitario y Politécnico La FE de Valencia, 46026 València, Spain; rafael.aloscompany@gmail.com; 7 Hospital de Manises, 46940 Manises, Spain; fran.navarro.vicente@gmail.com

**Keywords:** Crohn’s disease, fistula, succinate

## Abstract

The pathogenesis of Crohn’s disease-associated fibrostenosis and fistulas imply the epithelial-to-mesenchymal transition (EMT) process. As succinate and its receptor (SUCNR1) are involved in intestinal inflammation and fibrosis, we investigated their relevance in EMT and Crohn’s disease (CD) fistulas. Succinate levels and SUCNR1-expression were analyzed in intestinal resections from non-Inflammatory Bowel Disease (non-IBD) subjects and CD patients with stenosing-B2 or penetrating-B3 complications and in a murine heterotopic-transplant model of intestinal fibrosis. EMT, as increased expression of Snail1, Snail2 and vimentin and reduction in E-cadherin, was analyzed in tissues and succinate-treated HT29 cells. The role played by SUCNR1 was studied by silencing its gene. Succinate levels and SUCNR1 expression are increased in B3-CD patients and correlate with EMT markers. SUCNR1 is detected in transitional cells lining the fistula tract and in surrounding mesenchymal cells. Grafts from wild type (WT) mice present increased succinate levels, SUCNR1 up-regulation and EMT activation, effects not observed in SUCNR1^−/−^ tissues. SUCNR1 activation induces the expression of Wnt ligands, activates WNT signaling and induces a WNT-mediated EMT in HT29 cells. In conclusion, succinate and its receptor are up-regulated around CD-fistulas and activate Wnt signaling and EMT in intestinal epithelial cells. These results point to SUCNR1 as a novel pharmacological target for fistula prevention.

## 1. Introduction

Crohn’s disease (CD) is a chronic inflammatory pathology of the gastrointestinal tract characterized by transmural inflammation, a disruption of the epithelial barrier function and a dysregulated immune response. Its relapsing nature often determines its evolution from the initial inflammatory phenotype into stricturing (B2) and/or penetrating (B3) behaviors characterized by the appearance of serious complications such as the formation of intestinal stenosis and/or fistulas [1]. Both structural abnormalities seem interrelated as more than 95% of intra-abdominal fistulas appear within or at the proximal end of a stricture [2,3]. About one-third of patients are affected by this secondary complication, which is not prevented by the available drugs [4]. This leaves surgery as the only therapeutic option, and post-chirurgical recurrence is frequently seen [5].

A fistula is an abnormal tract connecting two surfaces covered by epithelial cells with a lumen filled with inflammatory cells, cell debris and erythrocytes [6]. The molecular mechanisms involved in fistula development are widely unknown, but reported evidence suggest that it implies the epithelial-to-mesenchymal transition process (EMT) [7,8], which normally contributes to the physiological tissue repair [3,9,10,11] and, in circumstances of deregulation, to fibrosis [12]. Through the EMT, epithelial cells show a progressive loss of their characteristic features with a concomitant acquisition of mesenchymal morphology, markers and function. Specifically, epithelial cells reduce the expression of the epithelial cell–cell adhesion molecule E-cadherin and lose the apical–basal polarity and the cell–cell junctions. In consequence, they increase their motility and, in parallel, express mesenchymal markers such as vimentin, fibroblast-specific protein-1 (FSP1), α-smooth muscle actin (α-SMA) and the profibrotic cytokine transforming growth factor-β (TGF-β) [13]. EMT is triggered by several signaling routes—TGF-β/Smad, Wnt, Notch and Hedgehog pathways—that converge in the activation of members of the SNAIL transcription factor family, e.g., Snail1 and Slug (Snail2) [14].

We have recently reported high levels of serum succinate and an increased expression of its receptor SUCNR1 (or GPR91) in surgical resections from CD patients and demonstrated the implication of this route in murine intestinal inflammation and fibrosis [15]. SUCNR1 is activated by succinate when this metabolite is secreted to the extracellular milieu after accumulation inside cells suffering metabolic alterations provoked by inflammatory mediators. This has been observed in a number of pathological conditions [16,17,18,19] and SUCNR1 has been detected in many cell types including macrophages, immature dendritic cells [20], fibroblasts and epithelial cells [15], all of them involved in CD complications.

In this study, we aimed to analyze whether succinate and SUCNR1 play a role in fistula formation by activating EMT in intestinal epithelial cells. Our results demonstrate for the first time that succinate and its receptor, which are significantly up-regulated in the fistula tract and the surrounding tissue, promote EMT through the activation of Wnt signaling in intestinal cells, which leads us to point to SUCNR1 as a potential target to prevent fistula development in CD patients.

## 2. Materials and Methods

### 2.1. Patients

Intestinal resections from CD patients were obtained after surgery. CD patients were subdivided according to Montreal classification into B2 with a stricturing behavior or B3 with a penetrating behavior (Table 1 and Table 2). The surgeons selected the intestinal samples from the stricture in B2-CD patients or from the area surrounding the fistula tract in B3-CD patients. In 5 B3-CD patients, the entero-enteric fistula specimens (fistula tract) and a sample close to the fistula tract (non-fistula tract) were obtained for immunohistochemistry analysis. As non-Inflammatory Bowel Disease (non-IBD) control samples, non-damaged mucosa of intestinal resections from colon carcinoma patients was used. The study was approved by the Institutional Review Board of the Hospital of Manises (Valencia), The Hospital of Sagunto (Valencia) and The University Hospital of Zurich (Switzerland). Written informed consent was obtained from all participating patients.

### 2.2. Mice

Sucnr1^−/−^ mice (9–12 weeks old, 20–25 g weight, kindly provided by Dr. Kenneth McCreath) were bred into a C57Bl/6 background. C57Bl/6 were used as wild type (WT) mice. All animals were maintained under specific pathogen-free conditions and were co-housed to reduce potential differences in microbiota. All protocols were approved by the institutional animal care and use committees of the University of Valencia, and all experiments were performed in compliance with the European Animal Research Law.

### 2.3. Induction of Intestinal Fibrosis by Heterotopic Transplant of Colonic Tissue

Intestinal fibrosis was induced in vivo using a heterotopic intestinal transplant model as previously described [15]. Briefly, small pieces of colon from WT or SUCNR1^−/−^ mice were subcutaneously transplanted into the dorsal neck region of WT recipient mice. After 7 days, recipient mice were sacrificed by neck dislocation and intestinal grafts were obtained. An adjacent segment of the colon from each donor was kept to be used as autologous control tissue (named as day 0 or non-transplanted tissue).

### 2.4. Cell Culture

HT29 cells (American Type Culture Collection, VA, USA) were cultured in McCoy’s Medium Modified (Sigma-Aldrich, Madrid, Spain) supplemented with 10% inactivated FBS, 100 U/mL penicillin, 100 μg/mL streptomycin and 2 mM L-glutamine. HT29 cells were treated with different concentrations of succinate (0, 0.1, 0.5, 1 or 5 mM) or TGF-β (5 ng/mL) for 48 h or 7 days depending on the experiment. In the experiments of 7 days, the medium was changed every two days. In some cases, HT29 cells were treated with the inhibitor of the Wnt pathway, XAV939 (1 µM, Sigma-Aldrich, Madrid, Spain) for 48 h.

### 2.5. Small Interfering (siRNA) Transfection

HT29 cells were transfected with 20 pmol of specific SUCNR1 siRNA (Invitrogen Life Technologies, Barcelona, Spain) using Lipofectamine-2000 (Invitrogen Life Technologies, Barcelona, Spain) according to the manufacturer’s instructions. The efficiency of transfection was determined by analyzing the *SUCNR1* mRNA expression by qPCR. Sixteen hours post-transfection, HT29 cells were treated with succinate as described above.

### 2.6. RNA Isolation and Real-Time Quantitative PCR (RT-qPCR)

Intestinal resections from CD or non-IBD patients or intestinal grafts from WT or SUCNR1^−/−^ mice were homogenated using the GentleMACS Dissociator (Miltenyi Biotech, Gladbach, Germany). Total RNA from human, mouse tissue and cells were obtained using the extraction kit (Illustra RNAspin Mini, GE HealthCare Life Science, Barcelona, Spain) according to the manufacturer’s instructions. For reverse transcription, cDNA was obtained with the Prime Script RT reagent Kit (Takara Biotechnology, Dalian, China). Quantitative PCR (qPCR) was performed with the Prime Script Reagent Kit Perfect Real Time (Takara Biotechnology, Saint-Germain-en-Laye, France) in a thermo cycler Light Cycler (Roche Diagnostics, Mannheim, Germany). Results were expressed as fold increase calculated as follows: change in expression (fold) = 2 − Δ(ΔCT) where ΔCT = CT (target) − CT (housekeeping) and Δ(ΔCT) = ΔCT (treated) − ΔCT (control). In all cases, the housekeeping gene used was β-actin. Specific primers were designed according to the sequences present in Tables S1 and S2.

### 2.7. Protein Extraction and Western Blot Analysis

Total and nuclear proteins from tissues and HT29 cells were obtained as previously described [21]. The expression of several proteins (Table S3) was analyzed by Western blot. Equal amounts of protein were loaded onto SDS/PAGE gels. Membranes were incubated with specific primary antibodies (Table S3) and with a peroxidase-conjugated anti-mouse IgG (Thermo Scientific, Rockford, IL, U.S.A., 1:2500) or anti-rabbit IgG (Thermo Scientific, 1:5000). The signal was detected using supersignal west pico chemiluminescent substrate (Thermo Scientific) and a LAS-3000 (Fujifilm, Barcelona, Spain). The Image Gauge version 4.0 software (Fujifilm) was used to quantify the protein expression by means of densitometry. Total protein data were normalized to β-actin while data of nuclear protein were referred to nucleolin.

### 2.8. Immunofluorescence and Confocal Microscopy

HT29 cells were fixed with 2% paraformaldehyde for 20 min and permeabilized with 0.1% Triton-X100 for 10 min. After that, HT29 cells were sequentially incubated with blocking solution (PBS with 10% serum and 1% BSA) at room temperature for 1 h, with primary antibodies anti-Vimentin (1:100, Abcam ab92547) or anti-E-Cadherin (1:100, ThermoFisher RA222618) at 4 °C overnight, and a secondary antibody (anti-mouse-FITC, 1:200, Invitrogen F2761 for E-Cadherin staining, or anti-rabbit-TexasRed, 1:200, ThermoFisher T2767 for Vimentin staining) for 45 min at room temperature. All nuclei were stained with Hoechst33342 (2 µM). Cells and intestinal grafts were visualized with the confocal microscope Leica TCS SP8, and pictures were taken with the software LASX (Leica Application Suite X).

### 2.9. Immunohistochemical Studies

Immunostaining for SUCNR1 was performed in 5 µm sections of paraffin-embedded colonic tissues obtained from the entero-enteric from B3-CD patients. Antigen retrieval was carried out with 10 mM sodium citrate buffer at pH 6.0 (Dako Target Retrieval Solution) for 20 min at 98 °C. After the inactivation of endogenous peroxidase and blocking the slides for 1 h at room temperature, intestinal tissues were incubated with the primary antibody Anti-GPR91 antibody (1:1000, PA5-337891) overnight at 4 °C. One to two drops of anti-rabbit Ig (Peroxidase) ImmPress Reagent kit/Vector were added as a secondary antibody, and samples were incubated 30 min at room temperature. Signal was developed with ImmPACT DAB Peroxidase substrate. Control negative was performed without primary antibody. All samples were counterstained with hematoxylin, and pictures were obtained with the Imager Z2 microscope (Zeiss) and the software AxioVision (Zeiss).

### 2.10. Double Immunohistochemistry

Double immunohistochemistry in fistula specimens was performed as previously described [22]. Briefly, after analyzing the protein expression of the first primary antibody (SUCNR1) with DAB, the slides were washed with PBS, and biotin and avidin were blocked with Dako Cytomation Biotin Blocking System (Dako). Then, tissues were blocked and incubated with the second primary antibody: Vimentin, Abcam, ab92547, 1:200 or CD206, Sigma, HPA004114, 1:200. The signal from the second primary antibody was developed with Vector Purple Kit (Vector Laboratories). The specificity of each immunostaining was confirmed by the absence of both primary antibodies. 

### 2.11. Succinate Quantification

Succinate levels in intestinal tissue from non-IBD and IBD patients or WT and SUCNR1^−/−^ grafts were quantified with the Succinate Assay Kit (ab204718, Abcam) according to the manufacturer’s instructions. Briefly, frozen tissues were homogenated with Ultraturrax in the Succinate Assay Buffer, the homogenates were centrifuged, and the supernatants were filtered with 10 kDa spin columns (ab93349, Abcam). Samples were then incubated with the Reaction Mix in 96-well plates during 30 min at 37 °C, and the absorbance at 450 nm was measured with the microplate reader SpectraMax Plus 384 (Molecular Devices). Concentration of succinate was calculated from the standard curve.

### 2.12. Statistical Analysis

Data were expressed as mean ± SEM and compared by a *t*-test for comparisons between two groups and one-way analysis of variance (ANOVA) with Tukey post hoc correction or Kruskal–Wallis with Dunn’s post hoc correction where appropriate for multiple comparisons. A *p-*value < 0.05 was considered to be statistically significant. The correlation between different data obtained in human samples was analyzed using Spearman’s correlation coefficient.

## 3. Results

### 3.1. SUCNR1 Mediates the EMT Associated with Intestinal Fibrosis In Vivo 

**Figure 1:**
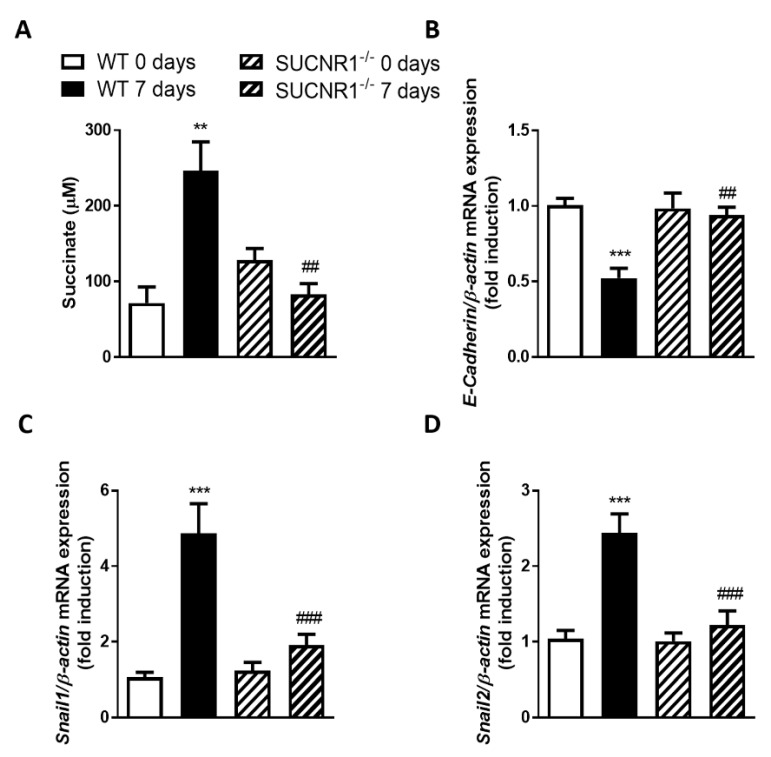
Lack of SUCNR1 decreases epithelial-to-mesenchymal transition (EMT) activation in vivo. Intestinal fibrosis was induced in vivo using the heterotopic transplant model. (**A**) Graph shows succinate levels in the intestinal grafts from wild type (WT) and SUCNR1^−/−^ mice (*n* = 4). (**B**–**D**) Graphs show the expression of *E-Cadherin, Snail1* and *Snail2* in intestinal grafts from WT and SUCNR1^−/−^ mice (*n* = 7). Bars in graphs represent mean ± SEM, and significant differences vs. intestinal grafts from WT at day 0 are shown by ** *p* < 0.01 and *** *p* < 0.001, and vs. intestinal grafts from WT at day 7 are shown by ## *p* < 0.01 and ### *p* < 0.001.

We analyzed the relevance of the SUCNR1 receptor in vivo with a heterotopic transplant model of intestinal fibrosis. Firstly, we observed that the levels of succinate in grafts from WT mice 7 days after transplantation were significantly higher than those in the autologous control tissue (day 0) (Figure 1A). These grafts also showed increased gene expression of the EMT markers Snail1 and Snail2 with a concomitant reduction on the expression of E-Cadherin (Figure 1B–D). In contrast, none of these changes was observed in the intestinal grafts from SUCNR1^−/−^ mice that, 7 days after transplantation, exhibited levels of succinate and of EMT markers’ gene expression similar to those observed in its autologous control tissue at day 0 (Figure 1B–D). Thus, our results demonstrate that the heterotopic transplant model induced the development of EMT, a process that is prevented in the absence of SUCNR1 receptor.

### 3.2. The intestinal Tissue Surrounding the Fistula Tract of B3-CD Patients Presents Increased Succinate Levels and an Overexpression of SUCNR1 that Correlates Positively with EMT Markers

**Figure 2:**
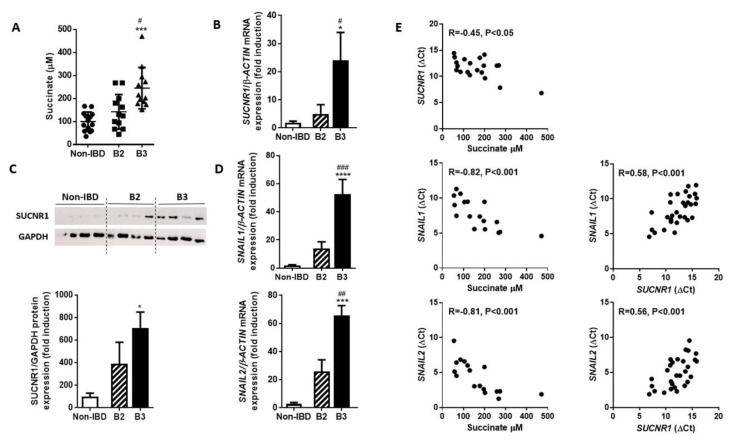
Intestinal succinate levels and SUCNR1 expression is increased in B3 CD-patients. Graphs show succinate levels (**A**), *SUCNR1* mRNA (**B**) and protein (**C**) expression, and the mRNA expression of *SNAIL1* and *SNAIL2* (**D**) in intestinal resections from non-Inflammatory Bowel Disease (non-IBD), B2 and B3 Crohn’s disease patients. In all cases, bars in graphs represent mean ± SEM, and significant differences vs. the non-IBD group are shown by * *p* < 0.05, *** *p* < 0.001 or **** *p* < 0.0001 and vs. B2 Crohn’s disease patients are shown by # *p* < 0.05, ## *p* < 0.01 or ### *p* < 0.001. (**E**) Correlation between data relative to the mRNA expression of *SUCNR1, SNAIL1,* and *SNAIL2* (expressed as delta Ct) and the levels of succinate on intestinal samples. A positive and significant correlation was detected between succinate levels and the expression of *SUCNR1, SNAIL1* and *SNAIL2* as well as between the expression of *SUCNR1* and that of *SNAIL1* and *SNAIL2*, (R = Spearman’s correlation coefficient).

The intestinal tissue that surrounds the fistula tract in B3-CD patients showed significantly higher levels of succinate and increased gene and protein expression of SUCNR1 in comparison with the values obtained in intestinal tissue from non-IBD patients or in fibrotic samples from B2-CD patients (Figure 2A–C). The expression of the transcription factors involved in EMT, SNAIL1 and SNAIL2, were also higher in samples from B3-CD patients than in tissues from non-IBD or B2-CD patients (Figure 2D). To analyze if these observations were interrelated, we analyzed the correlation between both pools of data and observed that the expression of SUCNR1 shows a positive and significant correlation with the expression of SNAIL1 and SNAIL2. On the other hand, the expression of both SUCNR1 and EMT transcription factors SNAIL1 and SNAIL2 positively correlated with succinate levels (Figure 2E). All these results suggest that succinate and its receptor SUCNR1 might be involved in the development of EMT in CD.

Given the association between inflammation and both the EMT process and the succinate-SUCNR1 pathway we sought to analyze the relationship between these elements in the intestinal samples included in this study. As expected, samples from B3-CD patients showed an increased gene expression of cytokines (IL6 and IL8) and of molecules expressed by antigen-presenting cells (CD86 and CD206) compared with control tissues (Figure S1A), suggesting a higher inflammatory activity in these tissues. Reinforcing the association between inflammation and succinate accumulation, a positive and significant correlation between succinate levels and the expression of cytokines was observed (Figure S1B). This relationship is further supported by the correlation observed between SUCNR1 and leukocyte markers’ expression in samples from B3 patients (Figure S1C).

### 3.3. SUCNR1 Is Expressed Specifically in the Fistula Tract

**Figure 3:**
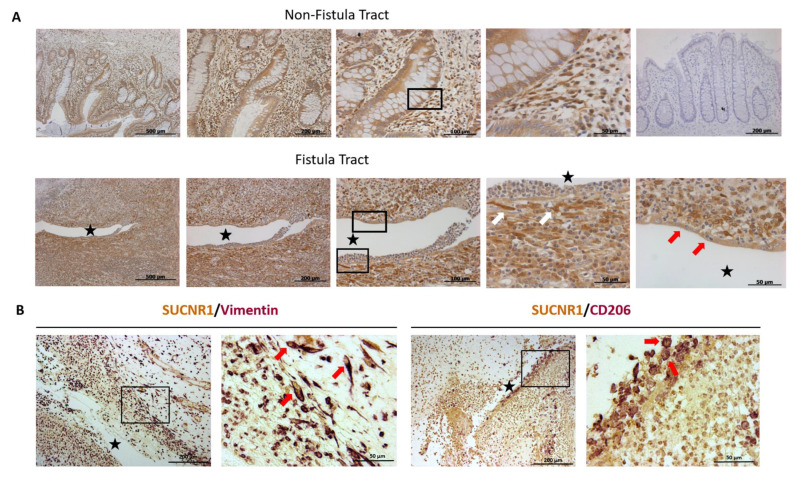
SUCNR1 is expressed in fibroblasts and macrophages of the fistula tract. (**A**) Immunostaining of SUCNR1 was performed in 5-µm sections of paraffin-embedded tissues, and representative pictures of areas without fistula (non-fistula tract) and areas including the tract of entero-enteric fistulas (fistula tract) from 5 B3-CD patients are shown. The black star indicates the fistula lumen. White arrows point to positive cells close to the fistula tract with a fibroblast morphology, and red arrows point to transitional cells (TCs). (**B**) Double immunohistochemistry of Vimentin-SUCNR1 and CD206-SUCNR1 was performed in 5-µm sections of paraffin-embedded tissues and representative pictures of areas including the fistula tract from 5 B3-CD patients are shown. Red arrows point to some cells double positive for SUCNR1/Vimentin or for SUCNR1/CD206.

A qualitative study of the expression of SUCNR1 in intestinal samples from B3-CD patients including the tract of entero-enteric fistulas and adjacent tissue was performed by immunohistochemistry. We observed that SUCNR1 is expressed in intestinal epithelial cells and in cells of the lamina propria. In all the entero-enteric fistulas analyzed, the intensity of the SUCNR1 staining was higher in cells close to the fistula tract than in more distant areas. SUCNR1 was specifically expressed in transitional cells (TC) lining this fistula tract (Figure 3A) and, according to the double immunostaining, in vimentin-positive cells (fibroblasts) and CD206-positive cells (probably macrophages) in the lamina propria around the fistula (Figure 3B).

### 3.4. Succinate Induces EMT through SUCNR1

**Figure 4:**
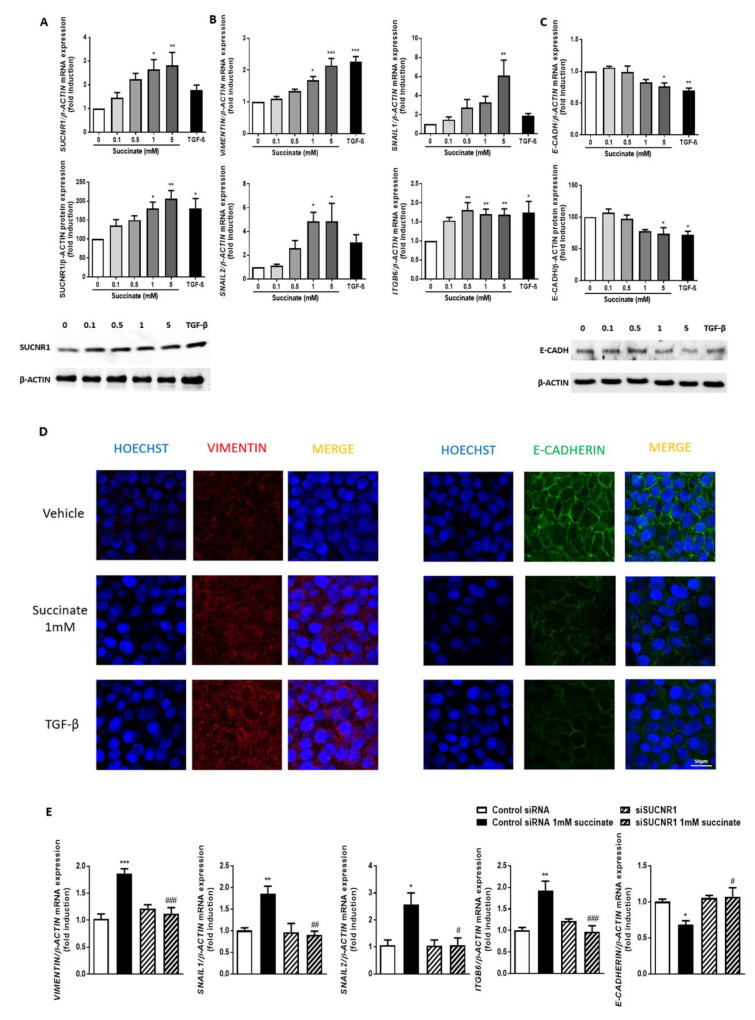
Succinate activates EMT in intestinal epithelial cells through SUCNR1 receptor. HT29 cells were treated with different concentrations of succinate (0, 0.1, 0.5, 1 and 5 mM) or TGF-β (5 ng/mL) for 48 h. (**A**) Graphs show the mRNA and protein expression of SUCNR1 receptor in HT29 cells (*n* = 5). The images correspond to a Western blot representative of a total of 5 independent experiments. (**B**) Graphs show the mRNA expression of the EMT markers, *VIMENTIN, SNAIL1, SNAIL2* and *ITGB6* in HT29 cells (*n* = 5). Bars in graphs represent mean ± SEM, and significant differences vs. non-treated HT29 cells are shown by * *p*< 0.05, ** *p* < 0.01 or *** *p* < 0.001. (**C**) Graphs show the mRNA and protein expression of E-CADHERIN in HT29 cells (*n* = 5). The image corresponds to a Western blot representative of a total of 5 independent experiments. (**D**) Immunofluorescence of VIMENTIN and E-CADHERIN in HT29 cells treated with 1 mM of succinate or TGF-β for 7 days. Representative pictures of a total of 3 independent experiments. (**E**) Graphs show the expression of *VIMENTIN, SNAIL1, SNAIL2, ITGB6* and *E-CADHERIN* in HT29 cells transiently transfected with a specific siSUCNR1 or with a control siRNA and treated with succinate 1mM for 48 h post-transfection. (*n* = 5). Bars in graphs represent mean ± SEM, and significant differences vs. control siRNA non-treated HT29 cells are shown by * *p* < 0.05, ** *p* < 0.01 and *** *p* < 0.001 and vs. siSUCNR1 non-treated HT29 cells are shown by # *p* < 0.05, ## *p* < 0.01 and ### *p* < 0.005.

We have previously reported that succinate induces the gene and protein expression of SUCNR1 in primary intestinal fibroblasts [15]. Hence, as a first strategy to detect whether the intestinal epithelial HT29 cells were responsive to this metabolite, we analyzed SUCNR1 expression after treatment with different concentrations of succinate (0, 0.1, 0.5, 1 and 5 mM) or its vehicle for 48 h. As shown in Figure 4A, treatment with succinate significantly increased the gene and protein expression of SUCNR1 in HT29 cells. Next, we analyzed whether these cells had activated the EMT process in response to succinate, and detected that succinate treatment had induced a significant and dose-dependent increase of the mRNA expression of mesenchymal (VIMENTIN) and EMT (SNAIL1, SNAIL2 and ITGB6) markers, with a concomitant reduction in the mRNA and protein expression of the epithelial marker E-CADHERIN (Figure 4B,C). In these experiments, we used TGF-β (5 ng/mL), as a positive control.

In line with this, immunofluorescence studies revealed a significant increase in the intensity of VIMENTIN immunostaining in HT29 cells treated with succinate or TGF-β. In parallel, both treatments reduced the staining intensity of E-CADHERIN. Moreover, a disturbed pattern of E-CADHERIN expression in the cytoplasmic membrane was specifically observed in succinate-treated cells (Figure 4D).

In order to investigate the role of SUCNR1 activation in the observed effects, we transiently knocked down this receptor using specific siRNAs constructs. We observed that HT29 cells transfected with specific SUCNR1 siRNA and treated with succinate 1mM failed to increase the expression of VIMENTIN, ITGB6, SNAIL1 and SNAIL2 and did not reduce the expression of E-CADHERIN (Figure 4E), which confirms that succinate induces EMT by stimulating SUCNR1.

### 3.5. SUCNR1 Stimulation by Succinate Activates the Wnt Pathway

**Figure 5:**
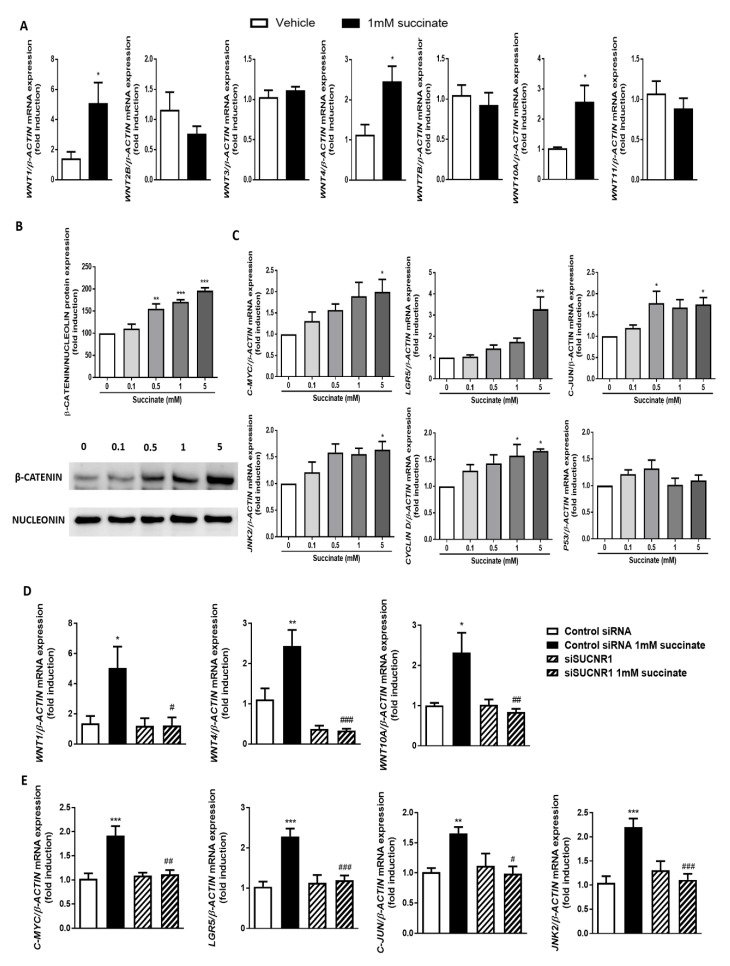
Succinate activates Wnt signaling pathway through SUCNR1 in intestinal epithelial cells. HT29 cells were treated with different concentrations of succinate (0, 0.1, 0.5, 1 and 5 mM) for 48 h. (**A**) Graphs show the expression of several Wnt ligands in HT29 treated cells. Succinate induces the expression of *WNT1, WNT4* and *WNT10A* (*n* = 5). Bars in graphs represent mean SEM and significant differences vs. non-treated HT29 cells are shown by * *p* < 0.05. (**B**) Nuclear proteins were isolated, and the expression of nuclear β-catenin was analyzed by Western blot. Graph shows the protein levels of nuclear β-catenin in HT29-treated cells, and the image corresponds to a Western blot representative of a total of 5 independent experiments. (**C**) Graphs show the expression of the Wnt target genes *C-MYC, LGR5, C-JUN, JNK2,* and *CYCLIN D* and of the tumor suppressor *P53* (*n* = 5). Bars in graphs represent mean ± SEM, and significant differences vs. non-treated HT29 cells are shown by * *p* < 0.05 or *** *p* < 0.001. (**D**) Graphs show the expression of *WNT1, WNT4* and *WNT10A* in HT29 cells transiently transfected with a specific siSUCNR1 or with a control siRNA and treated with succinate 1mM for 48 h post-transfection. (*n* = 5). Bars in graphs represent mean ± SEM, and significant differences vs. control siRNA non-treated HT29 cells are shown by * *p* < 0.05, ** *p* < 0.01 or *** *p* < 0.001, and vs. siSUCNR1 non-treated HT29 cells are shown by # *p* < 0.05, ## *p* < 0.01 and ### *p* < 0.005. (**E**) Graphs show the expression of the Wnt target genes *C-MYC, LGR5, C-JUN* and *JNK2* in HT29 cells transiently transfected with a specific siSUCNR1 or with a control siRNA and treated with succinate 1 mM for 48 h post-transfection. (*n* = 5). Bars in graphs represent mean ± SEM, and significant differences vs. control siRNA non-treated HT29 cells are shown by ** *p* < 0.01 or *** *p* < 0.001 and vs. siSUCNR1 non-treated HT29 cells are shown by # *p* < 0.05, ## *p* < 0.01 and ### *p* < 0.001.

Since it is widely assumed that Wnt signaling contributes to EMT activation and fistula formation, we sought to elucidate the influence of succinate and its receptor in this molecular pathway. For this purpose, we treated HT29 cells with different concentrations of succinate and analyzed different components of this route: the expression of Wnt ligands, the signaling pathway that implies the nuclear translocation of β-catenin and, finally, the expression of the most well-known target genes. First, from all the Wnt ligands described until this moment, we analyzed the effects of succinate on the expression of those that are easily detected (Ct values < 30) in HT29 cells. Treatment with 1 mM of succinate for 48 h induced a significant increase in the expression of WNT1, WNT4 and WNT10A, whereas no changes were detected in the expression of WNT2B, WNT3, WNT7B and WNT11 (Figure 5A). As shown in Figure 5B, succinate also increased the amount of nuclear Β-CATENIN in a dose response manner and, in line with this, induced the expression of the target genes (C-MYC, LGR5, C-JUN, JNK2 and CYCLIN D), without affecting that of p53 (Figure 5C).

To elucidate whether SUCNR1 mediates this activation of the Wnt pathway, we analyzed the effects of succinate in cells with a transient knock down of this receptor. Interestingly, succinate failed to increase the expression of Wnt ligands (Figure 5D) and of the target genes in siSUCNR1-transfected HT29 cells (Figure 5E). Therefore, as a whole, our results demonstrate that succinate induces the expression of Wnt ligands and activates the Wnt pathway in intestinal epithelial cells through SUCNR1.

### 3.6. Wnt Pathway Mediates Succinate-Induced EMT

**Figure 6:**
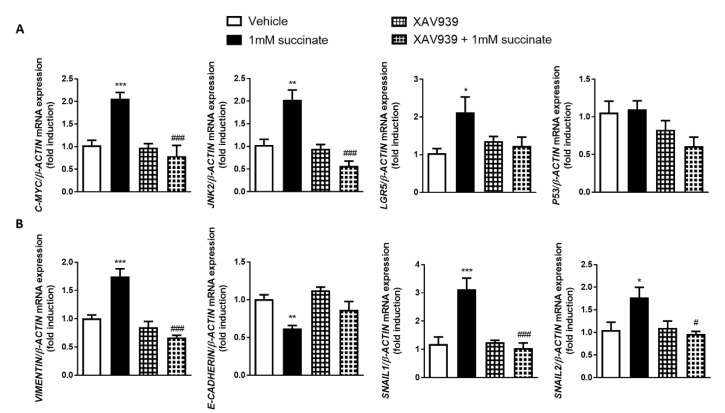
Succinate activates EMT through Wnt signaling pathway in intestinal epithelial cells. HT29 cells were treated with 1 mM of succinate and with the inhibitor of the Wnt pathway, XAV939 (1 µM) during 48 h. (**A**) Graphs show the expression of the Wnt target genes *C-MYC, JNK2, LGR5* and of the tumor suppressor *P53* (*n* = 5). (**B**) Graphs show the expression of the EMT markers *VIMENTIN, E-CADHERIN, SNAIL1* and *SNAIL2* (*n* = 5). Bars in graphs represent mean ± SEM, and significant differences vs. non-treated HT29 cells are shown by * *p* < 0.05, ** *p* < 0.01 or *** *p* < 0.001, and vs. HT29 treated with 1 mM of succinate are shown by # *p* < 0.05 or ### *p* < 0.005.

To analyze whether the activation of Wnt pathway was involved in the EMT activation induced by succinate, HT29 cells were co-treated with the Wnt inhibitor XAV939 1 µM or its vehicle for 48 h. As shown in Figure 6A, the increase in the expression of the Wnt-target genes C-MYC, LGR5 and JNK2 induced by succinate in vehicle-treated HT29 cells was significantly prevented by the presence of XAV939 (Figure 6A). Of interest, XAV939 treatment completely reverted the significant increase in the expression of the EMT markers VIMENTIN, SNAIL1, SNAIL2 and ITGB6 induced by succinate (Figure 6B). Accordingly, the reduction in the expression of E-CADHERIN induced by succinate was not observed in HT29 cells treated with the Wnt inhibitor XAV939.

## 4. Discussion

The present study demonstrates increased succinate levels in intestinal tissue from CD patients with a penetrating behavior and a high SUCNR1 expression in the fistula tract. Succinate, acting through SUCNR1, increases the expression of Wnt ligands, activates Wnt signaling and, consequently, provokes EMT. The essential role played by this process in fistula development and the correlation of succinate levels and SUCNR1 expression with the expression of EMT markers in B3-CD patients suggests the implication of the succinate-SUCNR1 pathway in this important complication of Crohn’s disease.

We have previously observed that succinate and its receptor are elevated in CD patients with a complicated disease (B2-B3) and that fibroblasts from these patients overexpress this receptor and respond to its activation with a profibrogenic pattern of expression [15]. Now, we observed that patients with a penetrating phenotype present higher levels of succinate and SUCNR1 than those presenting a stricturing disease and detected that the presence of this receptor is particularly enhanced in the fistula tract and its surroundings. Since succinate is a significant inducer of the expression of its own receptor, it seems reasonable to deduce that some local alterations around the fistula tract increase the concentration of the metabolite and, consequently, SUCNR1 expression. In fact, a positive and significant correlation was observed between succinate levels and SUCNR1 expression.

Samples from B3-patients present higher inflammatory activity than control and stenotic tissues, and, in coherence with the recognized association between metabolism and immune response [23], succinate levels positively correlate with cytokine expression. Additionally, the up-regulation of this route may also depend on the activity of TGF-β, key mediator in fistula formation that stimulates myofibroblasts with a concomitant increase in succinate release [24]. Finally, it may also be a consequence of the bacteria colonizing the fistula tract as it is a natural metabolic end product in some intestinal bacteria [25] and fistulas are often associated with infection [8]. In these areas of elevated succinate concentration, SUCNR1 expression is seen in most cellular types around the fistula, including cells lining the fistula tract, mesenchymal cells and innate immune cells. We observed that SUCNR1 expression correlates with that of the leukocyte markers CD86 and CD206, further substantiating the link between inflammation and the succinate-SUCNR1 pathway.

A fistula represents a tract between two surfaces that is lined by squamous epithelial cells or myofibroblast-like cells (“transitional cells”). The limited knowledge of its pathogenesis includes the implication of the EMT process [7,8], in which epithelial cells acquire a mesenchymal cell shape and exhibit enhanced motility and cell spreading, probably to make up for the reduced ability of fibroblasts to migrate and heal the injured mucosa observed in this subtype of CD patients [26]. We observed that transitional cells of the fistula tract express SUCNR1 and detected a positive and significant correlation between the expression of SUCNR1 and that of the EMT markers in the tissue around the fistula. These observations led us to infer that this receptor might be involved in this transitional process.

The coexistence of increased levels of succinate and EMT has been previously observed [27]. In fact, a complex and bidirectional instrumental relationship between alterations in cell metabolism and EMT has been described specially in the neoplastic context, where succinate dehydrogenase (SDH) activity is often hindered by different mechanisms [28]. SDH inhibition causes succinate accumulation, but whether succinate or its receptor are causally involved in the EMT process has not been analyzed. The heterotopic transplant model of intestinal fibrosis reproduces the accumulation of succinate and the occurrence of EMT in the presence of inflammation observed in B3-CD tissues. The absence of SUCNR1 prevents EMT, implicating the receptor in this process, but also the other two inter-related elements (succinate accumulation, in this study, and inflammation [15]), which makes it difficult to determine which is the primary action and which are the consequential secondary effects. However, EMT markers did not correlate with the expression of inflammatory cytokines in B3-CD-affected tissues, and more importantly, our in vitro experiments clearly demonstrate that succinate and its receptor directly induce EMT in intestinal epithelial cells. We demonstrated that succinate stimulates SUCNR1 and puts into action the Wnt signaling cascade, which is an essential player in EMT activation [29]. We observed that succinate increases the expression of some canonical Wnt ligands (Wnt1, Wnt4 and Wnt10a), promotes the nuclear translocation of β-catenin (the key element of the canonical Wnt signaling) and stimulates the expression of the most well-known target genes of this pathway (c-myc, cjun, Lgr5 and cyclinD) in intestinal epithelial cells. Very little is known regarding the transcription factors involved in the expression of Wnt ligands, but one possibility is that succinate stabilizes HIF-1 [30,31], which promotes the expression of Wnt1 ligand [32] and induces EMT in intestinal epithelial cells through the transcription factor ZEB1 [33]. However, HIF-1 stabilization by succinate in macrophages is only partially dependent on SUCNR1 [30], while our results show that both the expression of Wnt ligands and the stimulation of EMT by succinate imply the succinate receptor since they are prevented when SUCNR1 is knocked down. Nonetheless, the implication of the Wnt pathway in succinate-induced EMT was demonstrated by its prevention by the Wnt inhibitor XAV939. Taking all these results together, we deduce that succinate activates SUCNR1 receptor, which in turn stimulates the Wnt pathway to trigger the activation of EMT in intestinal epithelial cells and, in this way, promotes their motility and migratory capacity. These results may be also important in the oncology context given the relevant role of EMT in cancer progression [34]. In fact, numerous studies reported succinate accumulation in malignancies such as familial paraganglioma/pheochromocytoma, thyroid cancer, neuroblastoma, ovarian and gastric cancers [35,36,37,38]. Promotion of EMT may add to other pro-cancerous actions, such as the induction of angiogenesis, stimulated by this metabolite [38].

Although some evidence of the ability of succinate to promote intestinal remodeling exists [39], the confirmation of the implication of this metabolite and its receptor in fistula development in vivo has been precluded by the lack of a reliable and reproducible animal model of intestinal fistulas [40]. SAMP1/YITFc mice spontaneously develop a chronic ileitis that, in a small portion of subjects, is accompanied by perianal fistulas [41]. However, it is unknown whether EMT contributes to the formation of these fistulas and, even if it was the case, we would not have a way to determine the possible contribution of SUCNR1 to this process. On the other hand, a recent study describes a fistula model consisting in the implantation of intestinal tissue from human fetus subcutaneously in mice, but it would not be useful to our aim because the formation of entero-cutaneous fistulas in these animals seems to rely specifically on the activation of cells of human origin [42], and therefore, knocking down SUCNR1 would not be possible.

In summary, this manuscript shows for the first time that fistulas from CD patients present a local up-regulation of succinate and its receptor and demonstrates that the activation of this receptor by succinate triggers the activation of EMT in intestinal epithelial cells through Wnt signaling pathway. These results identify SUCNR1 as a potential pharmacological target to prevent fistula development in CD patients.

## Figures and Tables

**Table 1:** Information of patient characteristics.

	B2-CD Patients	B3-CD Patients	Non-IBD Patients
**Number of Patients**	19	16	10
**Age**			
Median	43	43	54
Interval	[22–77]	[15–61]	[28–76]
**Gender**			
Male	6	5	4
Female	13	11	6
**Localization**			
Terminal Ileum	9	7	4
Cecum	6	6	-
Colon	4	3	6

**Table 2:** Information of patient treatments.

Treatment	B2-CD Patients	B3-CD Patients
Azathioprine	2	1
Azathioprine + 5-aminosalicylic acid	1	0
Infliximab	6	5
Adalimumab	1	3
Adalimumab + Azathioprine	4	2
Adalimumab + Methotrexate	1	0
Ustekinumab	0	3
Ustekinumab + Infliximab	1	0
Vedolizumab	1	0
Certolizumab	2	2

## Supplementary Materials

The following are available online at https://www.mdpi.com/2073-4409/9/5/1104/s1, Figure S1: The expression of inflammatory mediators is increased in B3 CD-patients, Table S1: Specific primers used for human genes, Table S2. Specific primers used for mouse genes, Table S3. Primary antibodies used in Western Blot.
