# Supplementary material for: Succinate Activates EMT in Intestinal Epithelial Cells through SUCNR1: A Novel Protagonist in Fistula Development
Source: Cells. 2020 Apr 29;9(5):1104. doi: 10.3390/cells9051104 (PMC7290938; doi:10.3390/cells9051104)
Supplement: Supplementary file 1 [file cells-09-01104-s001.pdf]

A

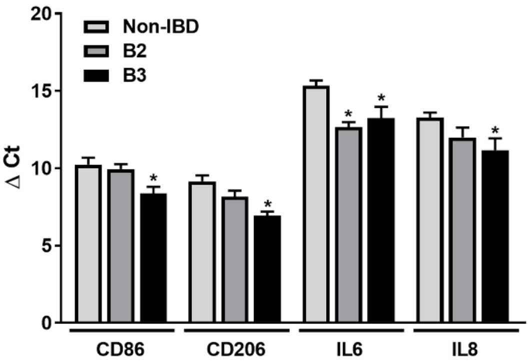

B

	SUCCINATE LEVELS
ΔCt CD86	-0,28 (0,14)
ΔCt CD206	-0,15 (0,45)
ΔCt IL6	**-0,55 (0,0015**)**
ΔCt IL8	**-0,40 (0,024*)**

C

B3 PATIENTS	ΔCt SUCNR1	ΔCt SNAIL1	ΔCt SNAIL2
ΔCt CD86	**0,63 (0,027*)**	-0,12 (0,71)	-0,34 (0,29)
ΔCt CD206	**0,78 (0,005**)**	-0,04 (0,91)	-0,20 (0,53)
ΔCt IL6	-0,33 (0,31)	-0,21 (0,53)	0,05 (0,88)
ΔCt IL8	-0,06 (0,86)	0,48 (0,12)	0,43 (0,16)

Figure S1. The expression of inflammatory mediators is increased in B3 Crohn's disease (CD) patients. (**A**) Graphs show the mRNA expression (in ΔCt) of macrophage markers (CD86 and CD206) and pro-inflammatory cytokines (IL6 and IL8) in intestinal resections from non-Inflammatory Bowel Disease (non-IBD), B2 and B3 Crohn's disease patients. In all cases, bars in graphs represent mean ± SEM, and significant differences vs. the non-IBD group are shown by $*p < 0.05$. (**B**) Table with the values of the Spearman's correlation coefficient R and the corresponding *p*-value between data relative to the mRNA expression of *CD86, CD206, IL6* and *IL8* (expressed as delta Ct) and the levels of succinate on intestinal samples. The expression of *IL6* and *IL8* shows a positive and significant correlation with succinate levels. (**C**) Table with the values of the Spearman's correlation coefficient R and the corresponding *p*-value between data relative to the mRNA expression of *CD86, CD206, IL6, IL8, SUCNR1, SNAIL1* and *SNAIL2* (expressed as delta Ct) on intestinal samples from B3-CD patients. The expression of *SUCNR1* shows a positive and significant correlation with that of *CD86* and *CD206.*
